# CTLA-4 Checkpoint Inhibition Improves Sepsis Survival in Alcohol-Exposed Mice

**DOI:** 10.4049/immunohorizons.2300060

**Published:** 2024-01-16

**Authors:** Cameron W. Paterson, Katherine T. Fay, Ching-Wen Chen, Nathan J. Klingensmith, Melissa B. Gutierrez, Zhe Liang, Craig M. Coopersmith, Mandy L. Ford

**Affiliations:** * Department of Surgery, Emory Critical Care Center, Emory University School of Medicine, Atlanta GA; † Lieutenant, Medical Corps, Naval Reserve Officer Training Corp, United States Navy, Atlanta, GA; ‡ Department of Surgery, Emory Transplant Center, Emory University School of Medicine, Atlanta GA

## Abstract

Chronic alcohol use increases morbidity and mortality in the setting of sepsis. Both chronic alcohol use and sepsis are characterized by immune dysregulation, including overexpression of T cell coinhibitory molecules. We sought to characterize the role of CTLA-4 during sepsis in the setting of chronic alcohol exposure using a murine model of chronic alcohol ingestion followed by cecal ligation and puncture. Results indicated that CTLA-4 expression is increased on CD4^+^ T cells isolated from alcohol-drinking septic mice as compared with either alcohol-drinking sham controls or water-drinking septic mice. Moreover, checkpoint inhibition of CTLA-4 improved sepsis survival in alcohol-drinking septic mice, but not water-drinking septic mice. Interrogation of the T cell compartments in these animals following pharmacologic CTLA-4 blockade, as well as following conditional *Ctla4* deletion in CD4^+^ T cells, revealed that CTLA-4 deficiency promoted the activation and proliferation of effector regulatory T cells and the generation of conventional effector memory CD4^+^ T cells. These data highlight an important role for CTLA-4 in mediating mortality during sepsis in the setting of chronic alcohol exposure and may inform future approaches to develop targeted therapies for this patient population.

## Introduction

CTLA-4 is a coinhibitory protein that plays an important role in downregulating T cell responses by competing with CD28 for CD80/CD86 ligands, inducing transendocytosis of CD80/CD86 to reducing the amount of these ligands available for costimulation of effector cells, and by directly attenuating TCR signal transduction by binding to TCRζ and inhibiting tyrosine phosphorylation after T cell activation (1–3). Manipulation of this pathway has been a focus of several immunomodulatory therapies including in cancer, where CTLA-4 blockade has been shown to limit tumor growth in various murine models and in several human cancers (4–7). Importantly, CTLA-4 is inducibly expressed on conventional T cells but is constitutively expressed on immunosuppressive regulatory T cells (Treg) (8). Given shared features of immune dysfunction, the translation of oncological immunomodulatory therapies to sepsis models has been a new and promising potential therapeutic strategy for a syndrome that affects 49 million people per year worldwide and accounts for 19.7% of world deaths, yet for which no therapy exists beyond antibiotics and supportive care (9). Although sepsis immunopathology initially involves dysregulated innate immune activation driving a maladaptive hyperinflammatory response, most patients will survive this initial phase and later develop widespread immune incompetence leaving them vulnerable to nosocomial and opportunistic pathogens (10–12). Multiple mechanisms contribute to this immune incompetence, including widespread cellular apoptosis (13) as well as upregulation of coinhibitory proteins on T cells. For example, CTLA-4 expression on CD4^+^ T cells has been correlated with both APACHE II and SOFA scores in septic patients (14). Similarly, in murine models of septic peritonitis, investigators have not only shown CTLA-4 expression to increase following cecal ligation and puncture (CLP), but also that administration of low-dose anti–CTLA-4 mAb improves sepsis mortality (15). Collectively, these data show that the CTLA-4 coinhibitory pathway is implicated in sepsis immunopathology and suggest that manipulation of this pathway using mAb therapies may translate to improved outcomes for septic patients.

CTLA-4 expression has also been shown to increase on Treg during sepsis (15), and this is associated with increased Treg suppressive capacity and impaired ability to clear infections such as *Salmonella* (16). However, the role of Treg in sepsis-induced immune dysfunction is complex. Several human studies have demonstrated increased circulation of Treg in sepsis and trauma patients (17–19) associated with increased mortality (17) and impaired T cell responses (20, 21). Murine studies have also shown the ability of Treg to suppress T cell proliferation (22) and dampen the host immune response during sepsis (23). Conversely, Treg have also been shown to exert protective effects (24) by mitigating sepsis-associated inflammatory organ insults such as acute lung injury (25) and have been shown to be necessary for host recovery following the initial acute inflammatory response (26). Treg can be categorized into central Treg (cTreg) versus effector Treg (eTreg) subsets, of which the latter has greater CTLA-4 expression and as a result has been targeted with depleting forms of anti-CTLA-4 mAb (27–29). In mice, eTreg are characterized as CD62L^lo^CD44^hi^ whereas cTreg show a CD62L^hi^CD44^lo^ phenotype (30–33) and also express Ly6C (34–37). Functionally, cTreg are quiescent and restricted to secondary lymphoid organs where they suppress T cell activation (34) and differentiate into eTreg following CD28 costimulation (31, 33, 35, 36). Conversely, eTreg are highly activated cells dependent on costimulatory molecules such as ICOS and CD28 for maintenance (31, 38, 39), and they express low Bcl2 (40–42) and high Ki67 (40), CD69 (34), and GITR (27). eTreg are potent suppressors of end-organ inflammation and migrate to inflamed target tissues through expression of homing molecules (29, 31, 38, 43) and can display polarized responses (e.g., Th1, Th2, Th17) similar to conventional effector T cells (Tconv) (33, 43).

However, effective translation of immunomodulatory therapies to sepsis must also account for other common comorbidities present in this patient population that may alter the immune response. Alcohol use disorder (AUD) is one such frequently encountered comorbidity that affects roughly 18.3 million individuals in the United States and 76.3 million individuals globally, with alcohol abuse accounting for 1.8 million deaths per year (44). Comorbid alcohol abuse can specifically complicate the care of critically ill patients, and patients with AUD have been shown to have more frequent intensive care unit admissions with longer stays and increased mortality (45). As it relates to sepsis, a 2016 study of diagnosis trajectories in 120,000 septic patients demonstrated that alcohol abuse carried a 2.2-fold increased risk of death compared with patients without comorbidities (46). Additionally, patients in the community with chronic alcohol use disorder are also more likely to initially develop sepsis both prior to and during a hospital admission (47, 48). Although it is clinically evident that patients with AUD experience worse outcomes from sepsis, the mechanisms by which this occurs are poorly elucidated. It is known that chronic alcohol use can lead to altered immune function. For example, in the innate immune system, murine models have shown chronic alcohol exposure can promote downregulation of CD80/CD86 expression on APC, thereby impairing their ability to activate T cells (49). In the adaptive immune system, chronic alcohol exposure has been associated with Th2 polarization and impaired T cell responses (50) as well as significant leukopenia in both rodents (51, 52) and humans (53) characterized by decreased naive T cells and homeostatic proliferation of polyclonal memory T cells by self-peptide rather than cognate Ag exposure (54–58). The effect of chronic alcohol exposure specifically on Treg is less clear; however, one study demonstrated reduced Treg and Th17/Treg imbalance within the dermis (59). Similarly, the role of T cell coinhibitory proteins in the setting of chronic alcohol exposure has yet to be fully explored. T cells of patients with alcohol-induced hepatitis have been shown to increase expression of coinhibitory markers PD-1 and T cell Ig and mucin domain 3 (TIM-3), and blockade of these proteins was found to restore T cell function (60); however, no studies have investigated the role of CTLA-4 in the immunologic alterations present during chronic alcohol exposure.

There have likewise been few investigations examining the immunologic sequalae of chronic alcohol exposure and sepsis in combination. Importantly, our laboratory has previously reported significantly worsened survival (61) as well as delayed expression of T cell activation markers (62) following CLP in chronically alcohol-exposed mice versus water-fed controls. However, the role of T cell coinhibitory molecules, specifically CTLA-4, in the setting of chronic alcohol exposure and sepsis has not been explored. Thus, in this study, we aimed to understand the role of CTLA-4 in chronically alcohol-exposed septic mice.

## Materials and Methods

### Animals

Six- to 8-wk-old C57BL/6 (B6) male and female mice were purchased from The Jackson Laboratory. This study was approved by the Emory University Institutional Animal Care and Use Committee (protocol PROTO201800161), and Institutional Animal Care and Use Committee guidelines were followed for animal care. Animals were given at least 2 wk acclimation prior to allocation to experimental groups and initiation of alcohol (EtOH) consumption. Isoflurane inhalation was performed for euthanasia prior to sample collection, and CO_2_ asphyxiation was performed at the conclusion of survival studies.

### *Cd4-Cre CTLA-4*^fl/fl^ and *Foxp3-Cre Ctla4*^fl/fl^ knockout mice

CD4CreER^T2^ mice carrying tamoxifen-inducible Cre recombinase under control of the CD4 promoter were purchased from The Jackson Laboratory and crossed with *Ctla4*^fl/fl^ animals, which were a gift of Dr. Shimon Sakaguchi (Osaka University, Osaka, Japan). A colony was established within the animal research facility at Emory University to generate both male and female *Cd4*-CreER^T2^ *Ctla4*^fl/fl^ animals with tamoxifen-inducible deletion of CTLA-4 on CD4^+^ T cells. CD4CreER^T2^ *Ctla4*^fl/WT^, Cre-negative *Ctla4*^/fl^, and wild-type (WT) B6 mice were used as controls.

*Ctla4*^fl/WT^ animals were crossed with *Foxp3*^YFP-Cre^ mice purchased from The Jackson Laboratory to create *Foxp3*^YFP-Cre^ *Ctla4*^fl/fl^ animals that demonstrate genetic deletion of CTLA-4 on Foxp3-expressing cells detectable by the presence of YFP-linked Cre driver. There is documented autoimmune-mediated lethality (63) of mice homozygous for this genotype by ∼7 wk of age (and in hemizgygous males owing to the X-linked nature of *Foxp3*), and thus we were unable to subject both hemizygous males and homozygous females to a 12-wk alcohol drinking protocol. As such, we employed a breeding strategy to generate female mice heterozygous for the *Foxp3*^YFP-Cre^ driver, which resulted in relatively equal numbers of both CTLA-4–intact and CTLA-4–deficient Treg within a given animal due to mosaic expression of the X-linked *Foxp3* gene (63). Therefore, all experiments utilizing *Foxp3*^YFP-Cre^ *Ctla4*^fl/WT^ mice consisted of only female animals with CTLA-4 deficiency on half of Foxp3-expressing cells.

### Chronic alcohol ingestion model

Animals were randomized to receive either a water or alcohol (200 proof ethanol, Decon Labs, UN1170) diet. Alcohol-drinking animals were administered increasing concentrations of alcohol in water solution from 0 to 20% (v/v) during 2 wk (5% for 5 d, 10% for 5 d, and 15% for 5 d). Alcohol-drinking animals were then maintained for 10 additional weeks (total alcohol exposure for all animals prior to experimental use 12 wk) on 20% alcohol with weekly solution replenishment, whereas water-drinking animals received a standard diet for the same duration. We have previously used this model of alcohol ingestion and have found that it does not impact liver histology, gut integrity, renal function (61, 64), or body weight (65), and it also achieves blood alcohol concentrations of 28 mg/dl, which physiologically mirrors the alcohol consumption of a chronic moderate drinker (65).

### Tamoxifen administration

Fifty micrograms of tamoxifen (Sigma-Aldrich, T5648) was suspended in 700 μl of alcohol (200 proof ethanol, Decon Labs, UN1170) and combined with 3.8 ml of autoclaved sunflower oil (Sigma-Aldrich, S5007) to create a 1 mg/100 μl solution and stored at −20°C. Then, 1.5 mg/150 μl was administered i.p. for 5 consecutive days to all animals in experiments, including *Cd4*-CreER^T2^ *Ctla4*^fl/fl^ mice, and CLP was performed 7 d after the final tamoxifen treatment.

### Sepsis model

Animals were subjected to CLP to model polymicrobial intra-abdominal sepsis. Anesthesia was induced with isoflurane and a single dose of buprenorphine (0.1 mg/kg) was administered preoperatively (s.c.) for analgesia. A 1-cm midline incision was made and the cecum was externalized from the peritoneal cavity and ligated with 4-0 silk ties ∼1 cm from its base and punctured twice with a 25G needle, and a small volume of stool was extruded. The cecum was then returned to the peritoneal cavity and the abdomen was closed with 4-0 silk suture followed by surgical adhesive for closure of the skin. Animals undergoing sham laparotomy underwent cecal evisceration only prior to abdominal closure. All animals received 1 ml of warmed sterile normal saline s.c. immediately postoperatively to replace intraoperative volume losses and were also s.c. administered ceftriaxone (25 mg/kg) and metronidazole (12.5 mg/kg) (in ∼200 μl of sterile normal saline) every 12 h for the first 48 h following CLP. Animals were monitored every 12 h until either sacrifice at 24 h for sample collection or sacrifice at 7 d for survival studies.

### Anti–CTLA-4 Ab treatment

Anti–CTLA-4 mAb (clone UC10-4F10-11, Bio X Cell, West Lebanon, NH) was combined with sterile PBS to create a 50 μg/150 μl solution. Then, 50 μg of anti–CTLA-4 mAb (or PBS vehicle control) was administered i.p. 6 h after CLP for flow cytometry studies and at 6 and 24 h after CLP for survival studies.

### Flow cytometric analysis

Animals were sacrificed and their spleens harvested and processed into single-cell suspensions at 24 h following CLP. Splenocytes were first treated with Fc blocking agent (TruStain FcX, BioLegend). Surface staining was performed using anti–CD4-Pacific Blue (RM4-5, BD Biosciences), anti–CD8-Pacific Orange (MCD0830, Invitrogen), anti–CD3-Alexa Fluor 700 (500A2, BD Biosciences), anti–CD8-BV605 (53-6.7, BioLegend), anti–CD4-BV650 (RM4-5, BioLegend), anti–CD3-BV786 (17A2, BioLegend), anti–CD44-BV711 (IM7, BioLegend), anti–CD69-Alexa Fluor 700 (H1.2F3, BioLegend), anti–CD4-BUV395 (GK1.5, BD Biosciences), anti–CD3-BUV496 (145-2c11, BD Biosciences), anti–CD8-BUV737 (53-67, BD Biosciences), anti–CD44-BUV805 (IM7, BD Biosciences), anti–Ly6C-BV510 (HK1.4, BioLegend), anti–CD103-BV605 (2E7, BioLegend), anti–CD69-BV650 (H1.2F3, BioLegend), anti–ICOS-BV711 (C398.4A, BioLegend), anti–GITR-BV786 (DTA-1, BD Biosciences), anti–CD62L-PE-Dazzle (MEL-14, BioLegend), anti–CD28-PE-Cy7 (E18, BioLegend), and anti-CD25-allophycocyanin-Cy7 (PC61, BioLegend).

Cells analyzed for intracellular/intranuclear expression were then fixed and permeabilized (Foxp3/transcription factor fixation/permeabilization concentrate and diluent, eBioscience). Intracellular/intranuclear staining was then performed using anti–Helios-PerCP-Cy5.5 (22F6, BioLegend), anti–CTLA-4-PE (UC10-4B9, BioLegend), anti–CTLA-4-allophycocyanin (BN13, BD Biosciences), anti–Foxp3-allophycocyanin (FJK-16s, eBioscience), anti–Ki67-Alexa Fluor 700 (16A8, BioLegend), and anti–Bcl2-eFluor 450 (10C4, eBioscience). AccuCheck counting beads (Thermo Fisher Scientific) were added to calculate absolute T cell numbers per spleen. LSR II and LSRFortessa flow cytometers (BD Biosciences) were used to collect samples. Data were analyzed using FlowJo v10.6 software.

### Statistical analysis

Prism v9.0 software (GraphPad Software, San Diego, CA) was used for all statistical testing. Outliers were identified and excluded using a Grubb test with α = 0.05. Data were tested for Gaussian distribution using either the D’Agostino–Pearson omnibus or the Shapiro–Wilk normality test with α = 0.05. Normally distributed data were compared using a two-tailed unpaired *t* test whereas nonnormal data were compared with a two-tailed Mann–Whitney *U* test for single pairwise analysis. Multiple comparisons testing was performed with one-way ANOVA or a Kruskal–Wallis test followed by either Tukey posttest or Dunn test for multiple comparisons. Survival data were compared using a log-rank test. Data are expressed as mean ± SEM. The significance level was set to *p *= 0.05.

## Results

### CTLA-4 checkpoint inhibition results in improved survival in alcohol-exposed septic mice but not water-fed septic mice

**FIGURE 1.**
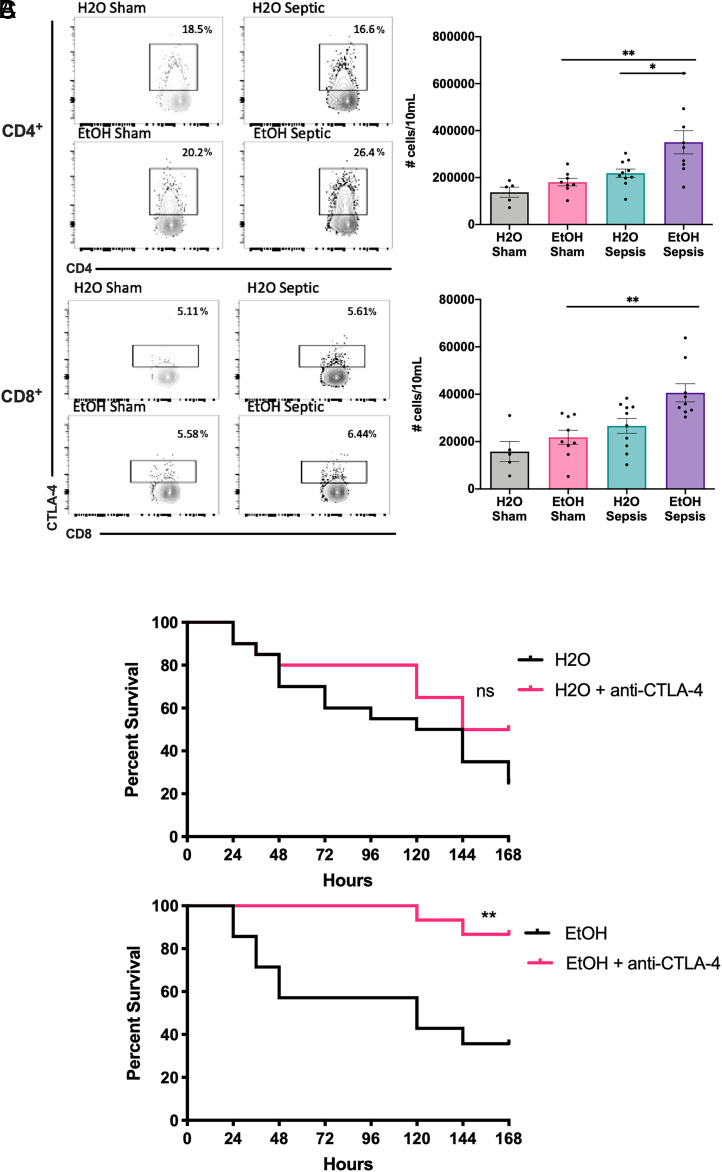
Alcohol-exposed septic mice have increased CTLA-4^+^CD4^+^ T cells relative to water drinkers and show a unique survival advantage in response to pharmacologic CTLA-4 checkpoint inhibition. Mice received either alcohol-infused or standard drinking water prior to being subjected to either sham surgery or CLP as described in *Materials and Methods*. For flow cytometry analysis, splenocytes were harvested at 24 h postoperatively. For survival studies, animals were treated with anti–CTLA-4 mAb at 6 and 24 h postoperatively and then monitored 7 d for survival. (**A**) Alcohol-exposed septic mice demonstrated increased numbers of CTLA-4–expressing CD4^+^ T cells (350,570 ± 49,412 cells/10 ml versus 218,506 ± 17,585 cells/10 ml, *p* = 0.01) but not CD8^+^ T cells (40,508 ± 3,812 cells/10 ml versus 26,617 ± 2,121 cells/10 ml, *p* = 0.14) versus septic water drinkers, as well as both increased CTLA-4^+^CD4^+^ T cells (35,0370 ± 49,412 versus 180,368 ± 16,124, *p* = 0.002) and CD8^+^ T cells (40,580 ± 3,812 cells/10 ml versus 21,809 ± 2,958 cells/10 ml, *p* = 0.006) versus alcohol-exposed sham animals. *n* = 5–10 mice/group. (**B**) Water-drinking septic mice treated with anti–CTLA-4 mAb showed no statistically significant improvement in survival compared with untreated controls (52 versus 40%, *p* = 0.39). *n* = 25 mice/group. (**C**) A unique survival advantage was found in alcohol-exposed septic mice treated with anti–CTLA-4 mAb versus untreated controls (86.7 versus 35.7%, *p* = 0.002). *n* = 14–15/group. Flow cytometry data displayed are from two independent experiments. Outliers were excluded using a Grubbs test (α = 0.05), and data were tested for normality and compared using either one-way ANOVA or a Kruskal–Wallis test followed by a Sidak or Dunn test for comparison of preselected groups and are displayed as mean ± SEM with representative flow cytometry plots shown to the left. Survival curves were compared using a log-rank test. *p < 0.05, **p < 0.01.

To interrogate the role of CTLA-4 coinhibition in chronically alcohol-exposed mice during sepsis, animals were randomized to 12 wk of either water or alcohol, then subjected to CLP or sham surgery and sacrificed 24 h later as described in *Materials and Methods* to assess splenic T cells. Although there were no differences in the number of CTLA-4–expressing CD4^+^ or CD8^+^ T cells isolated from water-fed versus alcohol-fed sham controls, alcohol-exposed septic mice exhibited a significant increase in the number of CTLA-4^+^CD4^+^ cells compared with water-drinking septic mice as well as alcohol-drinking sham controls (Fig. 1A). Evaluation of the CD8^+^ T cell compartment revealed no significant differences in the number of CTLA-4^+^ cells between septic groups; however, there were more CTLA-4^+^CD8^+^ cells in alcohol-fed septic mice compared with alcohol-fed sham controls (Fig. 1A). In light of this observed increase in CTLA-4 expression on CD4^+^ T cells in alcohol-drinking mice, we then tested the efficacy of anti-CTLA-4 mAb on 7-d survival following CLP in alcohol-drinking septic mice. Water-drinking mice given anti–CTLA-4 mAb exhibited no statistically significant survival difference following CLP as compared with untreated water-drinking controls (Fig. 1B). However, in mice chronically exposed to alcohol, administration of anti–CTLA-4 mAb after sepsis induction conferred a significant survival advantage versus untreated alcohol-drinking controls (Fig. 1C).

### Deletion of CTLA-4 specifically on CD4^+^ T cells improves survival in alcohol-exposed septic mice

**FIGURE 2.**
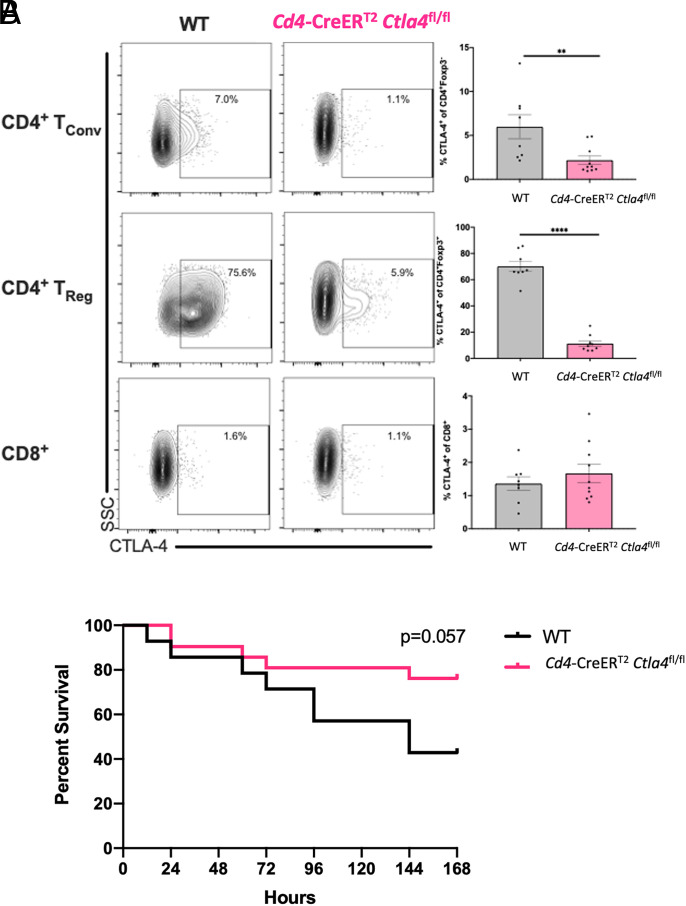
Deletion of CTLA-4 on CD4^+^ T cells alone improves survival in alcohol drinking septic mice. WT B6 and *Cd4*-CreER^T2^ *Ctla4*^fl/fl^ tamoxifen-inducible knockout mice received 12 wk of an alcohol diet and were then treated with tamoxifen prior to CLP, after which either splenocytes were collected at 24 h postoperatively or animals were monitored for 7-d survival as described in *Materials and Methods*. (**A**) Left, Representative flow cytometry plots from a single experiment with *n* = 8–10 mice/group showing frequency of CTLA-4 expression for WT versus *Cd4*-CreER^T2^ *Ctla4*^fl/fl^ animals in the CD4^+^ Tconv, CD4^+^ Treg, and CD8^+^ T cell compartments. Right, In alcohol-exposed septic *Cd4*-CreER^T2^ *Ctla4*^fl/fl^ mice, the frequency of CTLA-4 expression was significantly decreased in CD4^+^ Tconv (6.0 ± 1.4% versus 2.2 ± 0.5%, *p* = 0.009) and CD4^+^ Treg (70.2 ± 4.0% versus 11.3 ± 2.1%, *p* < 0.0001) but not in CD8^+^ T cells (1.1 ± 0.1% versus 1.7 ± 0.3%, *p* = 0.40) relative to septic WT alcohol-drinking animals. (**B**) *Cd4*-CreER^T2^ *Ctla4*^fl/fl^ septic alcohol drinking animals demonstrated a trend toward increased 7-d survival versus WT alcohol-drinking septic controls (76 versus 43%, *p* = 0.057). Data were pooled from two independent experiments with a total of *n* = 14–21 mice/group. For analysis of flow cytometry data, outliers were excluded using a Grubbs test (α = 0.05), and data were tested for normality and compared with either an unpaired *t* test or Mann–Whitney *U* test and are displayed as mean ± SEM. Survival data were analyzed with a log-rank test. **p < 0.01, ****p < 0.0001.

Given the observed survival benefit of anti–CTLA-4 in alcohol-drinking, but not water-drinking, septic animals, we sought to identify the cellular source of CTLA-4–mediating sepsis pathogenesis in alcohol-drinking mice. To begin to address this, we first sought to determine whether deletion of CTLA-4 specifically on CD4^+^ T cells would improve survival in alcohol-exposed septic mice. We crossed mice that express tamoxifen-inducible Cre recombinase under the control of the CD4 promoter (CD4CreER^T2^) with *Ctla4*^fl/fl^ mice to generate *Cd4*-CreER^T2^ *Ctla4*^fl/fl^ mice, which have tamoxifen-inducible deletion of CTLA-4 exclusively on all CD4^+^ T cells. Measurement of CTLA-4 expression in the CD4^+^Foxp3^−^ Tconv (CD4^+^ Tconv), CD4^+^Foxp3^+^ Treg (CD4^+^ Treg), and CD8^+^ T cell compartments of these mice after alcohol exposure and tamoxifen administration followed by CLP verified decreased CTLA-4 expression on both CD4^+^ Tconv and CD4^+^ Treg during sepsis (Fig. 2A). CTLA-4^+^ cells were essentially undetectable among CD8^+^ T cells in both WT and *Cd4*-CreER^T2^ *Ctla4*^fl/fl^ animals (Fig. 2A). Alcohol-drinking WT and *Cd4*-CreER^T2^ *Ctla4*^fl/fl^ mice were then monitored for survival following sepsis (Fig. 2B).

### CD44^−^CD62L^+^ cTreg are decreased in anti–CTLA-4 mAb-treated and *Cd4*-CreER^T2^ *Ctla4*^fl/fl^ conditional knockout alcohol-exposed septic mice

**FIGURE 3.**
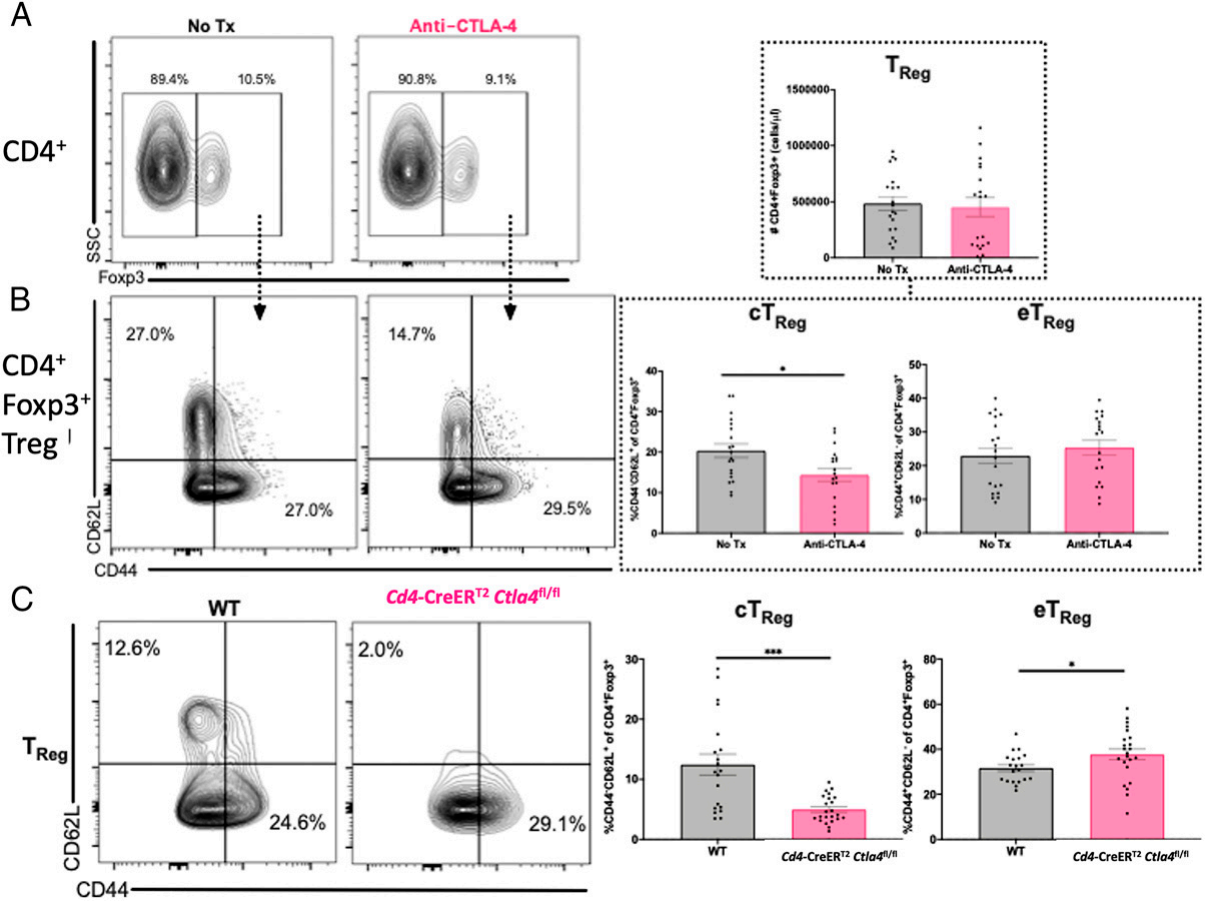
Anti-CTLA-4 mAb and CD4-specific CTLA-4 deficiency result in decreased cTreg in alcohol-exposed septic mice. (**A** and **B**) WT mice received 12 wk of an alcohol diet followed by anti-CTLA-4 mAb versus vehicle 6 h post-CLP and splenocytes were then harvested 24 h later. (A) Anti-CTLA-4 mAb did not alter the number of CD4^+^ Treg in alcohol drinking septic mice (483941 ± 61304 cells/μl versus 452551 ± 85577 cells/μl, *p* = 0.53). (B) Anti-CTLA-4 mAb decreased the frequency of CD44^-^CD62L^+^ cTreg in alcohol exposed septic mice (20.4 ± 1.7% versus 14.4 ± 1.6%, *p* = 0.01). The frequency of CD44^+^CD62L^−^ eTreg demonstrated a small increase in alcohol drinking septic mice (25.3 ± 2.2% versus 22.9 ± 2.3%, *p* = 0.46), but this was not statistically significant. Data displayed from 3 independent experiments with total *n* = 19-20 mice/group. Outliers were excluded using Grubbs test (α=0.05) and data were tested for normality and compared with either unpaired *t* test or Mann–Whitney *U* test and are displayed as mean ± SEM. (**C**) WT and *Cd4*-CreER^T2^ *Ctla4*^fl/fl^ tamoxifen-inducible knockout mice received 12 wk of an alcohol diet and were then treated with tamoxifen prior to CLP, and splenocytes were harvested 24 h postoperatively as described in *Materials and Methods*. Left, Representative flow cytometry plots demonstrating cTreg and eTreg frequencies in WT and *Cd4*-CreER^T2^ *Ctla4*^fl/fl^ alcohol-exposed septic mice. Right, Frequency of cTreg was decreased among alcohol-exposed septic *Cd4*-CreER^T2^ *Ctla4*^fl/fl^ mice (5.0 ± 0.5% versus 12.4 ± 1.8%, *p* = 0.0002) relative to WT animals, whereas the frequency of eTreg was increased (37.7 ± 2.4% versus 31.7 ± 1.5%, *p* = 0.046). Data displayed are from three independent experiments with a total of *n* = 20–23 mice/group. Outliers were excluded using a Grubbs test (α = 0.05), and data were tested for normality and compared with either an unpaired *t* test or Mann–Whitney *U* test and are displayed as mean ± SEM. *p < 0.05, ***p < 0.001.

Given our data showing that anti–CTLA-4 mAb confers a survival benefit to alcohol-exposed septic mice, we next interrogated changes in T cell compartments in anti–CTLA-4-treated alcohol-drinking septic mice to begin to understand the mechanistic basis for these findings. Although we found no difference in the overall number of CD4^+^ Treg following anti–CTLA-4 mAb administration to alcohol-drinking septic mice (Fig. 3A), the frequency of CD4^+^Foxp3^+^CD44^−^CD62L^+^ cTreg (34) was decreased following anti–CTLA-4 mAb treatment (Fig. 3B). No change in the frequency of CD4^+^Foxp3^+^CD44^+^CD62L^−^ eTreg was observed. The frequencies of CD4^+^Foxp3^+^CD44^−^CD62L^−^ Treg were not different between the groups (data not shown).

We next examined changes in the proportions of CD4^+^ Treg subtypes in alcohol-exposed *Cd4*-CreER^T2^ *Ctla4*^fl/fl^ conditional knockout animals. Similar to alcohol-drinking septic mice treated with anti–CTLA-4 mAb, *Cd4*-CreER^T2^ *Ctla4*^fl/fl^ knockout animals, which lack CTLA-4 exclusively within their CD4^+^ T cell compartment, exhibited decreased cTreg frequency relative to WT animals (Fig. 3C). Furthermore, the *Cd4*-CreER^T2^ *Ctla4*^fl/fl^ mice demonstrated a significantly increased frequency of CD4^+^Foxp3^+^CD44^+^CD62L^−^ eTreg (Fig. 3C). The frequencies of CD4^+^Foxp3^+^CD44^−^CD62L^−^ Treg were not different between the groups (data not shown). Taken together, these results demonstrate that blockade or deletion of CTLA-4 skews the Foxp3^+^ Treg compartment away from cTreg and toward eTreg.

### cTreg display increased activation and proliferation following deletion of CTLA-4 on CD4^+^ T cells in alcohol-exposed septic mice

**FIGURE 4.**
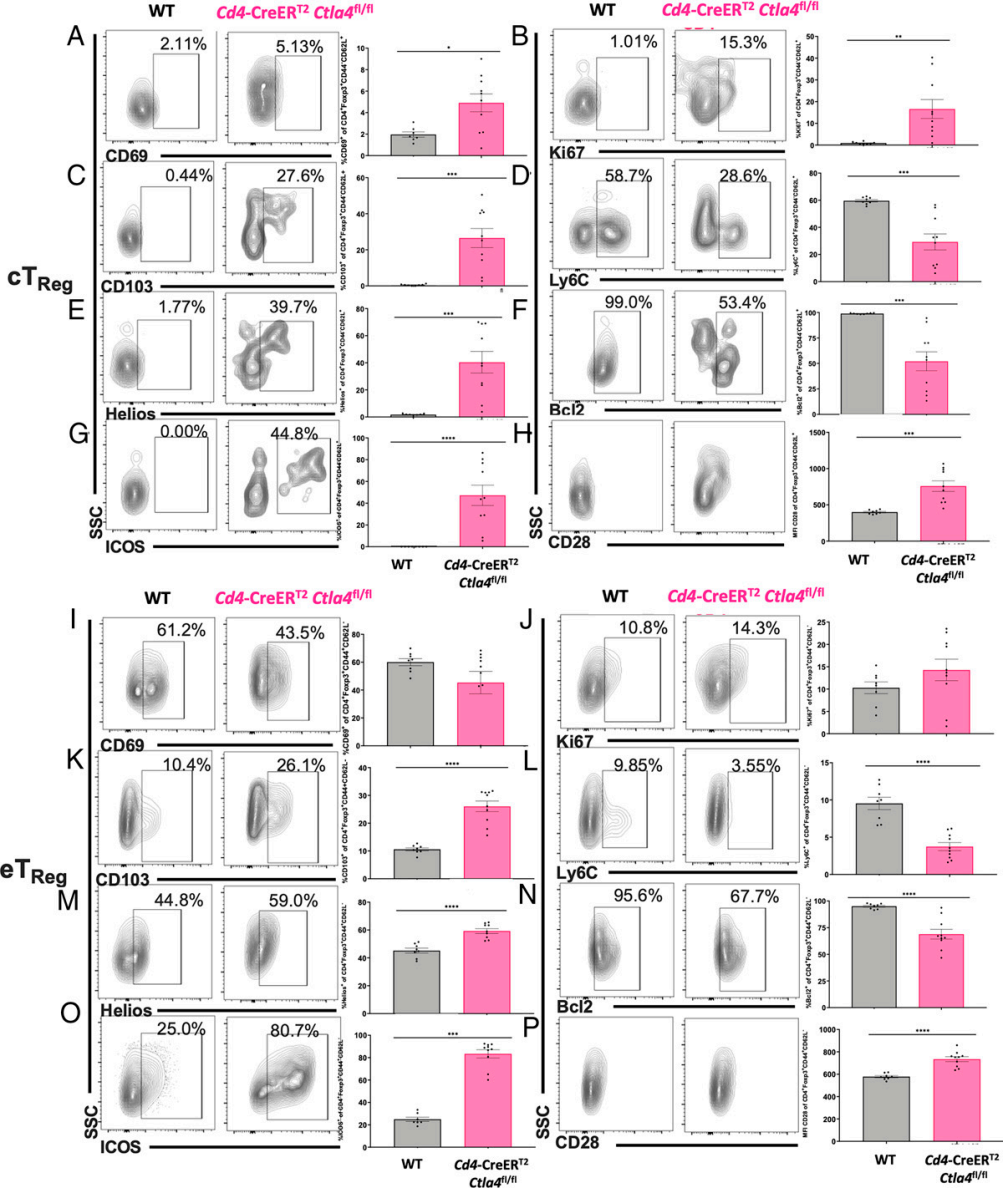
cTreg display increased activation following deletion of CTLA-4 on CD4^+^ T cells in alcohol-exposed septic mice. WT B6 and *Cd4*-CreER^T2^ *Ctla4*^fl/fl^ tamoxifen-inducible knockout mice received 12 wk of an alcohol diet and were treated with tamoxifen prior to CLP as described in *Materials and Methods*. Splenocytes were collected at 24 h and analyzed by flow cytometry. cTreg were defined as CD4^+^Foxp3^+^CD44^lo^CD62L^hi^ cells and eTreg were defined as CD4^+^Foxp3^+^CD44^hi^CD62L^lo^ cells as shown in Fig. 3. (**A**–**H**) CD4^+^Foxp3^+^CD44^lo^CD62L^hi^ cTreg were assessed for expression of CD69, CD103, Helios, ICOS, Ki67, Ly6C, Bcl2, and CD28. Representative flow cytometry plots are displayed on the left; summary data from *n* = 7–9 mice/group are displayed on the right. (**I**–**P**) CD4^+^Foxp3^+^CD44^hi^CD62L^lo^ eTreg were assessed for expression of CD69, CD103, Helios, ICOS, Ki67, Ly6C, Bcl2, and CD28. Representative flow cytometry plots are displayed on the left; summary data from *n* = 7–9 mice/group are displayed on the right. Outliers were excluded using a Grubbs test (α = 0.05), and data were tested for normality and compared with either an unpaired *t* test or Mann–Whitney *U* test and are displayed as mean ± SEM. *p < 0.05, **p < 0.01, ***p < 0.001, ****p < 0.0001.

We next sought to elucidate the impact of CTLA-4 deletion on cTreg and eTreg activation, proliferation, and differentiation in alcohol-drinking septic *Cd4*-CreER^T2^ *Ctla4*^fl/fl^ conditional knockout mice. In alcohol-exposed septic mice, deletion of CTLA-4 on CD4^+^ T cells (*Cd4*-CreER^T2^ *Ctla4*^fl/fl^) resulted in increased activation of CD4^+^Foxp3^+^CD44^−^CD62L^+^ cTreg as compared with WT cTreg, as evidenced by increased frequencies of CD69-, CD103-, and ICOS-expressing cells and increased CD28 mean fluorescence intensity (Fig. 4A–H). CTLA-4 deletion on CD4^+^ T cells also resulted in increased cTreg proliferation, as evidenced by increased expression of Ki67 (Fig. 4B), and increased cTreg stability, as evidenced by increased expression of the Helios transcription factor (Fig. 4E). In contrast, deletion of CTLA-4 on CD4^+^ T cells resulted in decreased frequency of Bcl2- and Ly6C-expressing cTreg (Fig. 4D, 4F).

We also examined the impact of CD4^+^ T cell–specific CTLA-4 deficiency on CD4^+^Foxp3^+^CD44^+^CD62L^−^ eTreg. These analyses showed an increase in the expression of costimulatory molecules CD28 and ICOS, an increase in Treg stability and homing molecules Helios and CD103, and a decrease in the frequency of Ly6C- and Bcl2-expressing cells in *Cd4*-CreER^T2^ *Ctla4*^fl/fl^ as compared with WT eTreg. In contrast to the effect observed in cTreg, *Cd4*-CreER^T2^ *Ctla4*^fl/fl^ eTreg exhibited a decrease in the frequency of CD69-expressing cells and no change in the frequency of Ki67^+^ proliferating cells (Fig. 4I–P).

### Targeted deletion of CTLA-4 results in increased eTreg via a cell-extrinsic mechanism in alcohol-exposed septic mice

**FIGURE 5.**
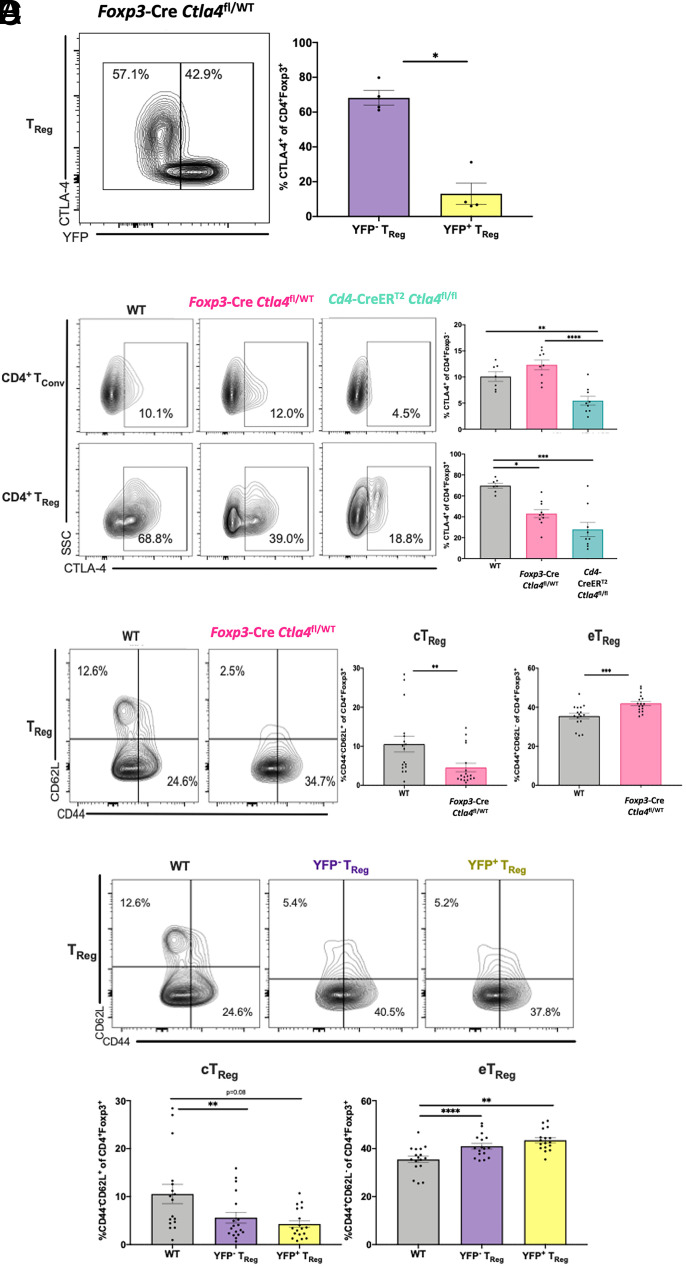
Conditional knockout of CTLA-4 specifically on Foxp3^+^ Treg results in the generation of eTreg via a cell-extrinsic mechanism. WT B6, germline *Foxp3*^YFP-Cre^ *Ctla4*^fl/WT^, and tamoxifen-inducible CD4CreER^T2^ *Ctla4*^fl/fl^ knockout mice received 12 wk of an alcohol diet. Mice in *Cd4*-CreER^T2^ *Ctla4*^fl/fl^ experiments were treated with tamoxifen prior to CLP. Splenocytes were collected at 24 h postoperatively. Representative flow cytometry plots are displayed to the left of figures. All data displayed were excluded for outliers using a Grubbs test (*p* = 0.05) and then tested for normality. Either an unpaired *t* test or Mann–Whitney *U* test was used for single pairwise comparisons, whereas either a one-way ANOVA or Kruskal–Wallis test followed by a Tukey or Dunn test was applied when multiple pairwise comparisons were made. All data are displayed as mean ± SEM. (**A**) Within Treg of *Foxp3*^YFP-Cre^ *Ctla4*^fl/WT^ mice, YFP^+^ Treg demonstrate significantly decreased CTLA-4 expression relative to YFP^−^ Treg within the same given animal (13.05 ± 6.07% versus 68.20 ± 4.20%, *p* = 0.03). Displayed data are from a single experiment with *n* = 4 mice/group. (**B**) *Cd4*-CreER^T2^ *Ctla4*^fl/fl^ mice demonstrate decreased frequency of CTLA-4 expression among CD4^+^ Tconv relative to WT (5.47 ± 0.84% versus 10.08 ± 0.92%, *p* = 0.006) and *Foxp3*^YFP-Cre^ *Ctla4*^fl/WT^ (5.47 ± 0.84% versus 12.32 ± 0.92%, *p* < 0.0001) animals. *Foxp3*^YFP-Cre^ *Ctla4*^fl/WT^ mice demonstrate decreased frequency of CTLA-4 expression among CD4^+^ Treg relative to WT animals (69.71 ± 2.38% versus 42.97 ± 3.78%, *p* = 0.03), but this was relatively less than that seen with *Cd4*-CreER^T2^ *Ctla4*^fl/fl^ animals (69.71 ± 2.38% versus 27.81 ± 6.79%, *p* = 0.48). Data displayed are from a single experiment with *n* = 7–10 mice/group. (**C**) The frequency of cTreg was decreased among alcohol-exposed *Foxp3*^YFP-Cre^ *Ctla4*^fl/WT^ (4.5 ± 1.1% versus 10.5 ± 2.0%, *p* = 0.006) septic mice relative to WT mice, whereas the frequency of eTreg was increased (42.0 ± 1.1% versus 35.5 ± 1.4%, *p *= 0.0008). Data are from three independent experiments with a total of *n* = 17–18 mice/group. (**D**) Both YFP^+^ Treg lacking CTLA-4 and YFP^−^ Treg with intact CTLA-4 expression possess decreased frequency of cTreg (4.3 ± 0.7% versus 10.5 ± 2.0%, *p *= 0.001; and 5.6 ± 1.1% versus 10.5 ± 2.0%, *p *= 0.08) and increased frequency of eTreg (43.5 ± 1.1% versus 35.5 ± 1.4%, *p *< 0.0001; and 41.0 ± 1.2% versus 35.5 ± 1.4%, *p *= 0.006) phenotypes compared with WT alcohol-drinking septic animals. Data are from three independent experiments with a total of *n* = 17–18 mice/group. *p < 0.05, **p < 0.01, ***p < 0.001, ****p < 0.0001.

We next sought to determine whether the observed impact of CD4-specific CTLA-4 deficiency on the composition of the Treg compartment in alcohol-drinking septic animals was due to the effect of CTLA-4 loss on CD4^+^ Treg alone without contribution from the accompanying loss of CTLA-4 on CD4^+^ Tconv that is present with both *Cd4*-CreER^T2^ *Ctla4*^fl/fl^ conditional knockout and CTLA-4 pharmacologic blockade. To test this hypothesis, we used a conditional knockout model resulting in deletion of CTLA-4 only on CD4^+^ Treg. Because Foxp3^YFP-Cre^ crossed with *Ctla4*^fl/fl^ animals to generate Treg-specific CTLA-4 conditional knockouts develop lethal autoimmunity at ∼7 wk of age as described (63), we were unable to subject them to a 12-wk alcohol protocol. To circumvent this issue, we examined female mice that were heterozygous for *Foxp3*-Cre (and *Ctla^fl/fl^*); in these animals, because *Foxp3* is X-linked, CTLA-4 is deleted on approximately half of Foxp3^+^ Treg due to the process of random X-inactivation in female cells (63). We first confirmed that in these *Foxp3*^YFP-Cre^ *Ctla4*^fl/WT^ animals, approximately half of CD4^+^Foxp3^+^ Treg demonstrated expression of the Cre transgene as detectable by fluorescence of YFP (Fig. 5A), and that whereas YFP^−^ Treg still exhibited high levels of CTLA-4 expression (Fig. 5A), YFP^+^ Treg exhibited an ∼5-fold reduction in the frequency of CTLA-4–expressing Treg (Fig. 5A). *Foxp3*^YFP-Cre^ *Ctla4*^fl/WT^ animals exhibited significantly reduced frequencies of CTLA-4^+^ Treg compared with WT mice, although not as low as those observed in tamoxifen-inducible CD4^CTLA-4 fl/fl^ knockouts (Fig. 5B). As expected, septic alcohol-exposed *Foxp3*^YFP-Cre^ *Ctla4*^fl/WT^ animals showed no difference in CTLA-4 expression versus WT mice among CD4^+^ Tconv, whereas *Cd4*-CreER^T2^ *Ctla4*^fl/fl^ knockout mice exhibited significantly decreased CTLA-4 among Foxp3^−^ T conv (Fig. 5B).

Using this model, we queried whether loss of CTLA-4 on Treg alone could recapitulate the reduced cTreg and increase eTreg observed in the CD4 conditional knockout model. We found that loss of CTLA-4 on Foxp3^+^ Treg was sufficient to result in decreased frequencies of cTreg and increased frequencies of eTreg in alcohol-exposed *Foxp3*^YFP-Cre^ *Ctla4*^fl/WT^ animals relative to WT mice (Fig. 5C). These results demonstrate that the deletion of CTLA-4 on Treg has an effect on the composition of the Foxp3^+^ Treg compartment in alcohol-drinking septic mice independently of the effect of deletion of CTLA-4 on Tconv. Finally, we sought to clarify whether the generation of eTreg following loss of CTLA-4 on Treg was occurring in an intrinsic manner by affecting the cells specifically on which CTLA-4 was lost, or in an extrinsic manner, in which the relative absence of CTLA-4 on ∼50% of Treg acted through a second-order mechanism to activate all Treg. Upon phenotypic comparison of Treg from WT versus *Foxp3*^YFP-Cre^ *Ctla4*^fl/WT^ animals, we found a decrease in cTreg and increase in eTreg in both CTLA-4–deficient (YFP^+^) and CTLA-4–intact (YFP^-^) cells within *Foxp3*^YFP-Cre^ *Ctla4*^fl/WT^ animals (Fig. 5D), suggesting that in the setting of chronic alcohol exposure and sepsis, CTLA-4 loss leads to activation of Treg via a cell-extrinsic mechanism.

### Deletion of CTLA-4 on Treg leads to the generation of CD4^+^ effector memory T cells in the conventional T cell compartment of alcohol-exposed septic mice

**FIGURE 6.**
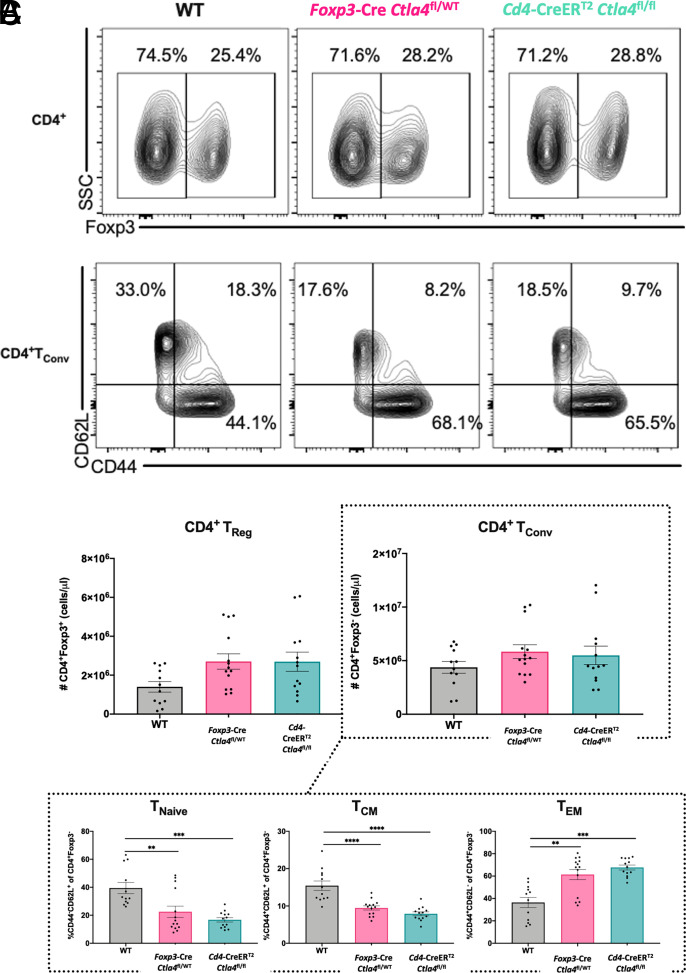
Deletion of CTLA-4 on Treg leads to the generation of CD4^+^ T_EM_ within the conventional T cell compartment of alcohol-exposed septic mice. WT B6, germline *Foxp3*^YFP-Cre^ *Ctla4*^fl/WT^, and *Cd4*-CreER^T2^ *Ctla4*^fl/fl^ tamoxifen-inducible knockout mice received 12 wk of an alcohol diet and were then treated with tamoxifen prior to CLP, after which splenocytes were collected at 24 h. (**A**–**C**) WT, *Foxp3*^YFP-Cre^ *Ctla4*^fl/WT^, and *Cd4*-CreER^T2^ *Ctla4*^fl/fl^ mice did not differ in number of CD4^+^ Treg or CD4^+^ Tconv. (B and C) Both *Foxp3*^YFP-Cre^ *Ctla4*^fl/WT^ and *Cd4*-CreER^T2^ *Ctla4*^fl/fl^ mice demonstrated decreased CD4^+^ T_naive_ (22.5 ± 4.1% versus 39.5 ± 4.0%, *p* = 0.007 and 16.7 ± 1.6% versus 39.5 ± 4.0%, *p* = 0.0006, respectively) and CD4^+^ T_CM_ (9.4 ± 0.6% versus 15.4 ± 1.3%, *p* < 0.0001 and 7.9 ± 0.5% versus 15.4 ± 1.3%, *p* < 0.0001, respectively) as well as increased CD4^+^ T_EM_ (61.4 ± 4.5% versus 36.4 ± 4.6%, *p* = 0.004 and 67.6 ± 2.2% versus 36.4 ± 4.6%, *p* = 0.0003, respectively) relative to WT animals. Representative flow cytometry plots are displayed on the left. Data are from two independent experiments with a total of *n* = 12–14 mice/group. Outliers were excluded using a Grubbs test (α = 0.05), and data were tested for normality and compared with either a one-way ANOVA or Kruskal–Wallis test followed by a Tukey or Dunn test and are displayed as mean ± SEM. **p < 0.01, ***p < 0.001, ****p < 0.0001.

Given that loss of CTLA-4 on Treg led to an effector CD4^+^ Treg phenotype via a cell-extrinsic mechanism in alcohol-exposed septic mice, we next queried whether such a mechanism would likewise lead to alterations within the CD4^+^ Tconv compartment as well. We first examined the numbers of CD4^+^ Tconv and CD4^+^ Treg in both *Foxp3*^YFP-Cre^ *Ctla4*^fl/WT^ and *Cd4*-CreER^T2^ *Ctla4*^fl/fl^ alcohol-exposed septic knockout animals and found no differences with respect to WT controls (Fig. 6A, 6C). However, within the CD4^+^ Tconv compartment, deletion of CTLA-4 on both 100 and 50% of CD4^+^ Treg led to decreased frequencies of CD4^+^ T_naive_ cells and CD4^+^ central memory T cells (T_CM_) as well as an increased frequency of CD4^+^ effector memory T cells (T_EM_) relative to WT controls (Fig. 6B, 6C).

### Deletion of CTLA-4 on CD4^+^ T cells leads to activation of CD4^+^ T_CM_ in alcohol-exposed septic mice

**FIGURE 7.**
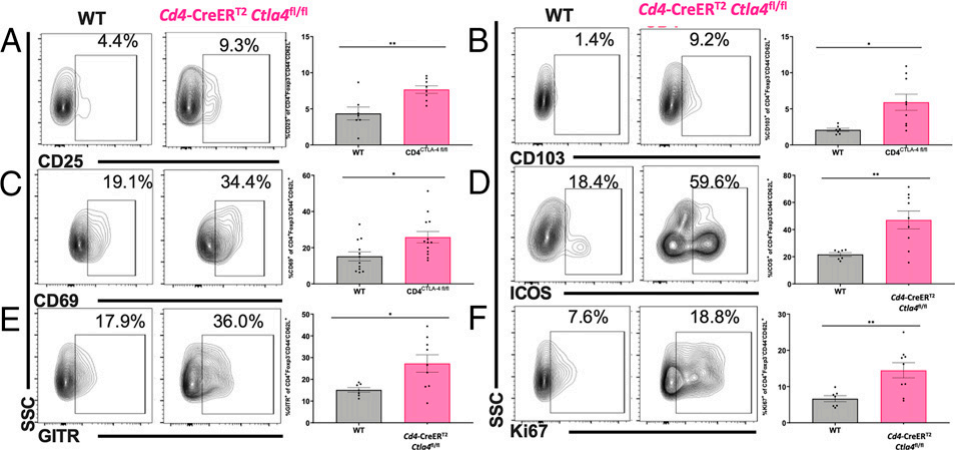
Deletion of CTLA-4 on CD4^+^ T cells increases markers of CD4^+^ T_CM_ activation. (**A**–**F**) WT and *Cd4*-CreER^T2^ *Ctla4*^fl/fl^ tamoxifen-inducible knockout mice received 12 wk of an alcohol diet and were treated with tamoxifen prior to CLP as described in *Materials and Methods*. Splenocytes were collected at 24 h for all animals. Relative to WT mice, CD4^+^ T_CM_ of *Cd4*-CreER^T2^ *Ctla4*^fl/fl^ mice demonstrated greater expression of CD25 (4.4 ± 0.9% versus 7.7 ± 1.5%, *p* = 0.006), CD69 (15.2 ± 2.5% versus 26.8 ± 3.1%, *p* = 0.02), GITR (15.1 ± 1.1% versus 27.2 ± 4.0%, *p* = 0.02), CD103 (2.1 ± 0.2% versus 5.9 ± 1.1%, *p* = 0.01), ICOS (21.7 ± 1.3% versus 47.1 ± 6.5%, *p* = 0.005), and Ki67 (6.6 ± 0.8% versus 14.5 ± 2.1%, *p* = 0.007). Representative flow cytometry plots are displayed on the left. Data displayed are from between one and two independent experiments with a total of *n* = 7–14 mice/group. Outliers were excluded using a Grubbs test (α = 0.05), and data were tested for normality and compared with either an unpaired *t* test or Mann–Whitney *U* test and are displayed as mean ± SEM. *p < 0.05, **p < 0.01.

Analogous to our observation of the increased activation status of cTreg following total deletion of CTLA-4 on CD4^+^ T cells, we next examined whether deletion of CTLA-4 on CD4^+^ T cells also increased markers of activation in CD4^+^ T_CM_ consistent with the generation of effector CD4^+^ Tconv. We found that total deletion of CTLA-4 on both CD4^+^ Tconv and CD4^+^ Treg using *Cd4*-CreER^T2^ *Ctla4*^fl/fl^ mice indeed resulted in significant increases in CD4^+^ T_CM_ expression of CD25, CD69, GITR, CD103, ICOS, and Ki67 among alcohol-exposed septic animals (Fig. 7).

### Anti–CTLA-4 mAb decreases CD4^+^ T_CM_ and increases CD4^+^ T_EM_ in alcohol-exposed septic mice

**FIGURE 8.**
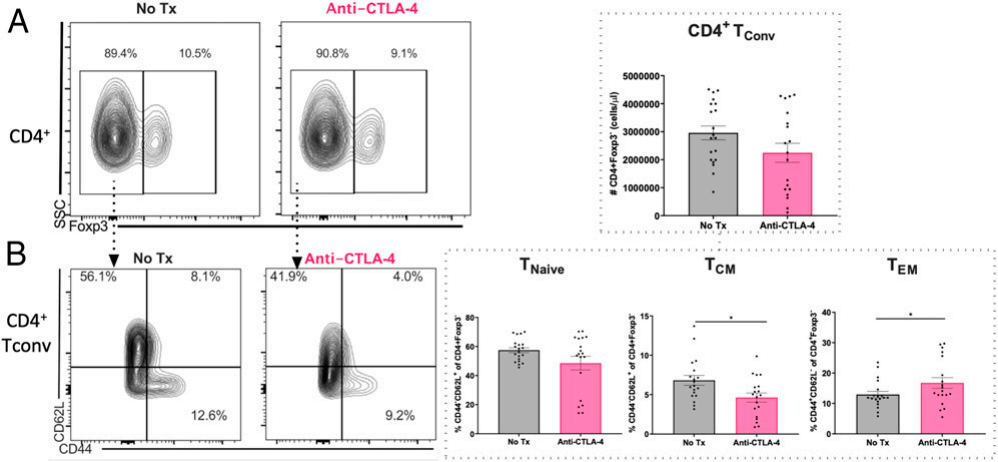
Anti–CTLA-4 mAb decreases CD4^+^ T_CM_ and increases CD4^+^ T_EM_ in alcohol-exposed septic mice. WT mice received 12 wk of an alcohol diet followed by anti–CTLA-4 mAb versus vehicle at 6 h after CLP, and splenocytes were then harvested 24 h later. (**A**) The number of CD4^+^ Tconv was not altered by anti–CTLA-4 mAb in alcohol-exposed septic mice relative to untreated animals (2,244,687 ± 340,404 cells/μl versus 2,957,554 ± 248,325 cells/μl, *p* = 0.10). (**B**) Relative to untreated alcohol-exposed septic animals, mice treated with anti–CTLA-4 mAb demonstrated a decreased frequency of CD4^+^ T_CM_ (4.6 ± 0.6% versus 6.8 ± 0.6%, *p *= 0.01) and increased frequency of CD4^+^ T_EM_ (16.7 ± 1.8% versus 12.9 ± 1.1%, *p *= 0.046) whereas the frequency of CD4^+^ T_naive_ cells (48.5 ± 4.6% versus 57.5 ± 1.7%, *p *= 0.46) was unchanged. Representative flow cytometry plots are show on the left. Outliers were excluded using a Grubbs test (α = 0.05), and data were tested for normality and compared with either an unpaired *t* test or Mann–Whitney *U* test and are displayed as mean ± SEM. Data are displayed from three independent experiments with a total of *n* = 19–20 mice/group. *p < 0.05.

Finally, we sought to verify whether the phenotypic changes in the form of increased effector CD4^+^ Tconv resulting from CTLA-4 deletion on Treg was also present in the setting of pharmacologic CTLA-4 blockade. We found no difference in the total numbers of CD4^+^ Tconv between untreated versus anti–CTLA-4 mAb-treated alcohol-exposed septic mice (Fig. 8A). However, similar to the findings observed in the conditional knockout mice, a significant decrease in the frequency of CD4^+^ T_CM_ and increase in the frequency of CD4^+^ T_EM_ among CD4^+^ Tconv was observed in alcohol-exposed septic mice treated with anti–CTLA-4 mAb (Fig. 8B).

## Discussion

Both sepsis and chronic alcohol use are known mediators of immune dysregulation characterized in part by increased coinhibitory marker expression (15, 60), yet little is known about the effects of alcohol on sepsis-induced immune dysregulation. Using a murine model of septic peritonitis in the context of chronic alcohol exposure, we investigated the T cell coinhibitory marker CTLA-4 and found an increase in its expression within the CD4^+^ T cell compartment. This increase in CTLA-4 is suggestive of decreased immune competence in alcohol-drinking septic mice as compared with either condition alone, or with healthy animals. Thus, in the context of the known excess morality in alcohol-fed septic mice (61), blockade of CTLA-4 presented a compelling therapeutic target to increase immune competence and decrease mortality under these conditions. We observed a significant survival advantage using anti–CTLA-4 mAb that was unique to alcohol-exposed septic mice, and similar results were obtained with deletion of CTLA-4 on CD4^+^ T cells alone. Interrogation of the Treg compartment using WT versus *Cd4*-CreER^T2^ *Ctla4*^fl/fl^ conditional knockout mice revealed a significant increase in the frequency of Ki67^+^ proliferating cTreg in the absence of CTLA-4 (Fig. 4B), but a decrease in the frequency of cTreg among total Treg (Fig. 2C). In contrast, we observed no significant increase in the frequency of Ki67^+^ proliferating eTreg in the absence of CTLA-4 (Fig. 4B), but observed an increase in the frequency of eTreg among total Treg (Fig. 2C). One hypothesis to explain these results is that proliferating Ki67^+^ cTreg may give rise to eTreg. An analogous activation of CD4^+^ T_CM_ and a shift to CD4^+^ T_EM_ was observed in the CD4^+^ Tconv compartment. These data suggest that loss of CTLA-4 in alcohol-exposed septic mice leads to increased immune activation, and thus could confer a survival advantage by increasing immune competence in alcohol-exposed septic animals.

We initially targeted CTLA-4 for checkpoint inhibition given data suggesting that this can facilitate a proinflammatory response to compensate for the relative immunoparalysis conferred by coinhibitory markers during sepsis (66–68). In particular, data showing a marked upregulation of CTLA-4 on CD4^+^ T cells isolated from alcohol-drinking mice suggest that the conventional cells are likely hyporesponsive and the Treg possess more potent suppressor activity. This is based on the known mechanism of action of CTLA-4 under homeostatic conditions (69), specifically its ability to sequester CD80/CD86 via transendocytosis (69). This function is cell-extrinsic and prevents APC CD80/CD86 from interacting with CD28 to activate T cells. Furthermore, the ability of global CTLA-4 deficiency to promote proliferation and effector function in the Tconv compartment has been reported previously in a variety homeostatic (70) and autoimmune (71, 72) conditions, while the ability of CTLA-4 deficiency on Treg alone to produce similar effects has also been previously documented in settings of lymphopenia (73) and autoimmunity (74). Additionally, our finding that CTLA-4–deficient Treg can induce activation of Tconv cells both reinforces existing literature as well as documents this phenomenon in a murine model of sepsis plus chronic alcohol exposure.

Our data show that deletion of CTLA-4 results in increased activated eTreg in the setting of sepsis. These findings are in line with published studies in CTLA-4–deficient mice in the absence of sepsis, which showed that despite the fact that they develop lethal lymphoproliferation with multiorgan inflammation, CTLA-4–deficient mice exhibit an increased population of proliferating Foxp3^+^ Treg (75, 76). However, CTLA-4–deficient Treg lack suppressor function and were unable to regulate disease in an adoptive transfer model of diabetes (75). Thus, the proliferation of Foxp3^+^ Tregs are negatively regulated by CTLA-4, but their suppressive function requires CTLA-4. Our data show that under conditions in which CTLA-4 is blocked, during which Treg proliferation is increased but suppressive function is inhibited, sepsis survival of alcohol-drinking mice is improved. From this, combined with the data showing that alcohol-drinking septic mice exhibit increased CTLA-4 expression on Treg compared with water-drinking septic mice, we infer that blocking CTLA-4 reduces Treg function and improves immune competence during sepsis. Moreover, in a study that addressed the role of eTreg in sepsis, Molinaro et al. (23) demonstrated that water-fed CCR4^−/−^ mice showed reduced Treg suppressive activity and improved survival during sepsis, which may suggest a harmful role for eTreg given their characteristic expression of CCR4 (33). Previous authors have reported that gastric cancer patients treated with anti–PD-1 mAb, a related checkpoint inhibitor, demonstrated proliferation of eTreg (27). Multiple authors have also reported the presence of an eTreg phenotype following both anti–CTLA-4 mAb (70, 77–80) and CTLA-4 deletion (72) under a variety of autoimmune and homeostatic conditions.

The finding that anti–CTLA-4 improves sepsis survival in alcohol-drinking but not water-drinking mice is an interesting and potentially unexpected observation. Inoue et al. (15) reported dose-dependent variation in the efficacy of anti–CTLA-4 in water-drinking septic animals, where a low dose (50 μg) was beneficial and a high dose (200 μg) was harmful to sepsis survival. Our results were generated using the lower dosing method, and thus it is possible that other factors such as differences in microbiota underlie the differences between the observations in our study compared with the Inoue et al. study. This hypothesis is consistent with observations in animals treated with anti–CTLA-4 in the setting of cancer, wherein studies showed that changes in intestinal microbiota altered susceptibility to anti–CTLA-4 checkpoint inhibition (81). In addition, our study and that of Inoue et al. used different clones of anti–CTLA-4 mAb. Moreover, we cannot eliminate the possibility that dose-dependent responses to anti–CTLA-4 mAb are further altered by chronic alcohol exposure. Excessive immune stimulation leading to adverse autoimmune events has previously been reported following anti–CTLA-4 treatment in melanoma patients (7), and therefore it is possible that the optimal therapeutic dose may vary between applications. Outside of the sepsis literature, studies showing that chronic alcohol exposure leads to downregulation of CD80/CD86 on APCs (49) in conjunction with our data showing differential CTLA-4 expression during chronic alcohol and sepsis in combination suggest that differences in the relative expression of CTLA-4, CD80/CD86, and CD28 between alcohol and water-fed mice may underlie unique responses between the two when these molecules are experimentally manipulated. Finally, it is also possible that exposure to alcohol could result in alterations in the intestinal microbiota, and thus secondarily impact resistance versus susceptibility via this mechanism. Future experiments to determine the impact of CTLA-4 blockade on the function and trafficking of both Treg and Tconv in the context of alcohol and sepsis will be beneficial in this regard.

We can conclude from our studies that the shifts in Treg and Tconv following *Ctla4* deletion are not dependent on either alcohol intake or sepsis alone, because the control groups are exposed to alcohol and are septic. However, we cannot conclude that the shifts in Treg and Tconv after *Ctla4* deletion in alcohol-exposed septic mice would not still occur in the absence of either alcohol exposure or sepsis. Even if they did, the observed changes are still relevant to the role of CTLA-4 in alcohol-drinking septic mice. The mechanism of action of anti–CTLA-4 mAb has also been disputed, particularly in cancer biology, with multiple studies now suggesting that the antitumor effects of some anti–CTLA-4 mAb variants primarily result from Ab-mediated depletion of CTLA-4–expressing Treg (82–84), which in effect predominantly depletes intratumoral eTreg given their high expression of CTLA-4 (27–29). Although we do not exclude this as a possible mechanism in our model, it is unlikely, as we found no changes to overall T cell numbers and only selective decreases of central T cell phenotypes in contrast with increased eTreg and CD4^+^ T_EM_ frequencies.

In summary, in this study, we have demonstrated that chronically alcohol-exposed septic mice exhibit increased expression of CTLA-4 on CD4^+^ T cells, and that subsequent loss of CTLA-4 specifically on Treg acts to promote a global shift to an effector phenotype among all CD4^+^ T cells, both conventional and regulatory. This Treg phenotypic shift is further associated with a survival advantage conferred by anti–CTLA-4 mAb in alcohol-drinking septic mice, which is not observed in water-fed septic mice. These data suggest that CTLA-4 checkpoint inhibition could be further investigated as a potential strategy to ameliorate sepsis-induced immune dysregulation specifically in patients experiencing chronic alcohol exposure.
